# Supplementary material for: Co-regulation and function of FOXM1/RHNO1 bidirectional genes in cancer
Source: eLife. 2021 Apr 23;10:e55070. doi: 10.7554/eLife.55070 (PMC8104967; doi:10.7554/eLife.55070)
Supplement: Supplementary file 1. [file elife-55070-supp1.docx]

**Supplementary File 1. *FOXM1* vs. *RHNO1* mRNA expression correlations.**

| Sample Type^1^ | Spearman r | P value | N | Analysis Method^2^ | Data Source |
| --- | --- | --- | --- | --- | --- |
| **HGSC tissues** | 0.819 | < 0.0001 | 36 | RT-qPCR | Current study |
| **OSE, FTE, and HGSC cell lines** | 0.779 | < 0.0001 | 15 | RT-qPCR | Current study |
| **Immortalized FTE cells (FT282)** | 0.583 | < 0.0001 | 1440 | scRNA-seq | Current study |
| **HGSC Cells (OVCAR8)** | 0.305 | < 0.0001 | 1455 | scRNA-seq | Current study |
| **Riken normal mouse tissues** | 0.663 | < 0.0001 | 169 | RNA-seq | Lizio et al, *Genome Biol*., 2015 |
| **GTEx normal tissues** | 0.595 | < 0.0001 | 9190 | RNA-seq | Vivian et al, *Nat Biotechnol*., 2017 |
| **Mouse intestinal epithelium** | 0.448 | < 0.0001 | 1522 | scRNA-seq | Haber, *Nature*, 2017 |
| **TCGA pan-cancer tissues** | 0.575 | < 0.0001 | 7814 | RNA-seq | Vivian et al, *Nat Biotechnol*., 2017 |
| **TCGA HGSC tissues** | 0.621 | < 0.0001 | 263 | RNA-seq | TCGA, *Nature*, 2011 |
| **Melanoma tissues** | 0.449 | < 0.0001 | 4645 | scRNA-seq | Tirosh, *Science*, 2016 |
| **HGSC tissues** | 0.595 | < 0.0001 | 29 | scRNA-seq | Winterhoff, *Gynecol Oncol*., 2017 |
| **CCLE pan-cancer cell lines** | 0.656 | < 0.0001 | 967 | Microarray | Barretina et al, *Nature*, 2012 |
| **CCLE HGSC cell lines** | 0.605 | 0.0022 | 23 | Microarray | Barretina et al, *Nature*, 2012 |
| **CCLE pan-cancer cell lines** | 0.657 | < 0.0001 | 1072 | RNA-seq | CCLE and GDSC, *Nature*, 2015 |
| **CCLE HGSC cell lines** | 0.756 | < 0.0001 | 21 | RNA-seq | CCLE and GDSC, *Nature*, 2015 |

**^1^ Samples are from human unless otherwise noted; OSE, ovarian surface epithelia; FTE, fallopian tube epithelia; GTEx, genotype tissue expression; TCGA, cancer genome atlas; CCLE, cancer cell line encyclopedia**

**^2^ RT-qPCR, reverse-transcriptase quantitative PCR; scRNA-seq, single cell RNA sequencing; RNA-seq, RNA sequencing**
